# Supplementary material for: The First Identification of the Uniqueness and Authentication of Maltese Extra Virgin Olive Oil Using 3D-Fluorescence Spectroscopy Coupled with Multi-Way Data Analysis
Source: Foods. 2020 Apr 15;9(4):498. doi: 10.3390/foods9040498 (PMC7230349; doi:10.3390/foods9040498)
Supplement: Supplementary file 1 [file foods-09-00498-s001.pdf]

*Supplementary Material.*

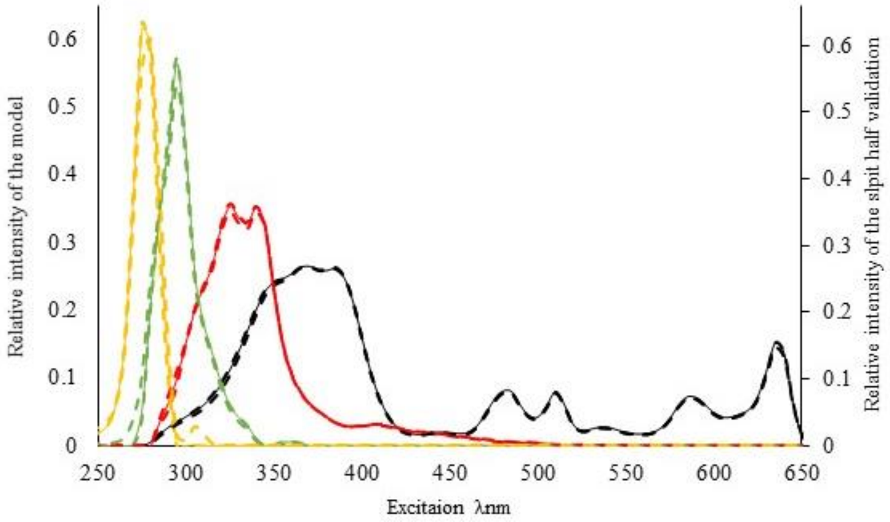


**Figure S1.** Mode 2 loadings (excitation) from non-negative constrained 4 component PARAFAC model, dotted lines represent components from split-half validation models. *1st component (Black), 2nd Component (Red), 3rd Component (Green) and 4th Component (Yellow).*

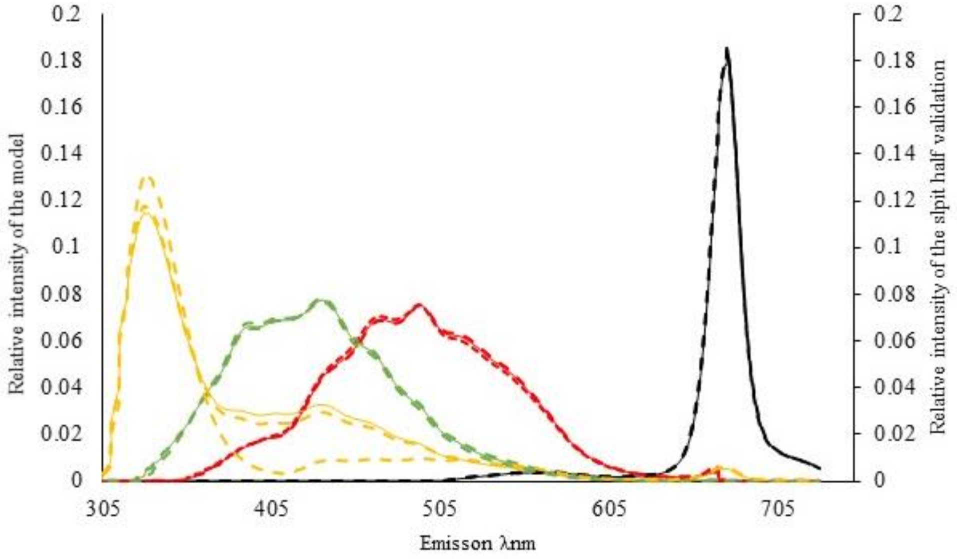


**Figure S2.** Mode 3 loadings (emission) from non-negative constrained 4 component PARAFAC model, dotted lines represent components from split-half validation models. *1st component (Black), 2nd Component (Red), 3rd Component (Green) and 4th Component (Yellow).*

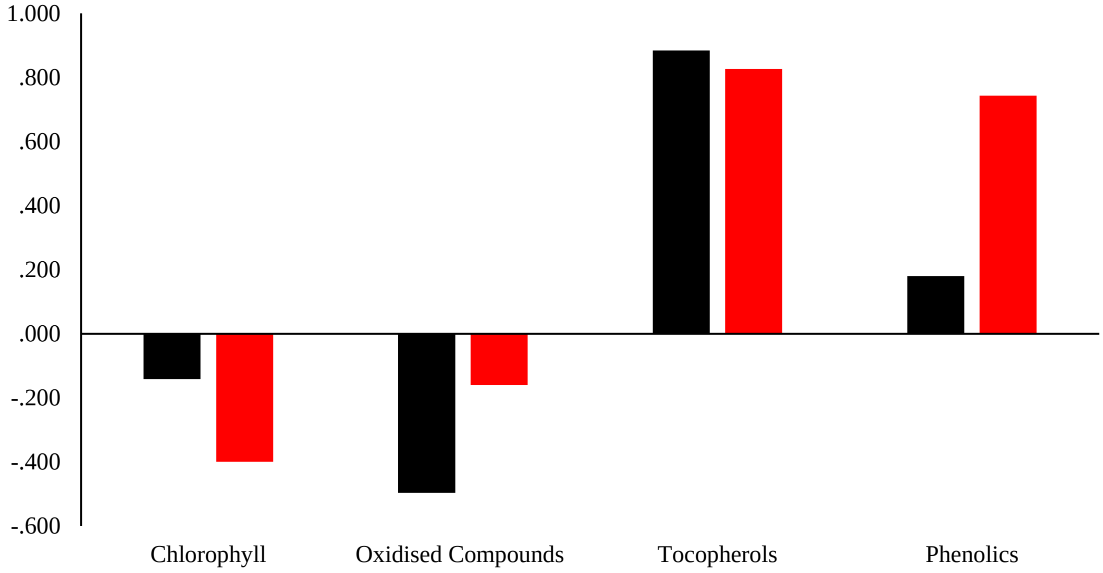


**Figure S3.** Standardized discriminant function coefficients (Black Bars) and Pearson's correlations of each variable with the discriminant function (Red Bars) obtained from the structure matrix.
